# ReShuffle-MS: Region-Guided Data Augmentation Improves Artificial Intelligence-Based Resistance Prediction in *Escherichia coli* from MALDI-TOF Mass Spectrometry

**DOI:** 10.3390/microorganisms14010177

**Published:** 2026-01-13

**Authors:** Dongbo Dai, Chenyang Huang, Junjie Li, Xiao Wei, Shengzhou Li, Qiong Wu, Huiran Zhang

**Affiliations:** 1 School of Computer Engineering and Science, Shanghai University, Shanghai 200444, China; dbdai@shu.edu.cn (D.D.); huangchenyang@shu.edu.cn (C.H.); lijunjie@shu.edu.cn (J.L.); xwei@shu.edu.cn (X.W.); 2 Department of Computer Science, University of Tsukuba, Tsukuba 305-8573, Japan; s2036012@u.tsukuba.ac.jp; 3 Department of Laboratory Medicine, Shanghai Sixth People’s Hospital Affiliated to Shanghai Jiao Tong University School of Medicine, Shanghai 200233, China

**Keywords:** antimicrobial resistance (AMR), data augmentation, machine learning, deep learning, mass spectrometry (MS), spectral recombination

## Abstract

Rapid antimicrobial resistance (AMR) prediction from MALDI-TOF mass spectrometry (MS) remains challenging, particularly when training artificial intelligence (AI) models under small-sample constraints. Performance is often hampered by the high dimensionality of spectral data and the subtle nature of resistance-related signals: full-spectrum approaches risk overfitting to high-dimensional noise, whereas peak-selection strategies risk discarding structurally informative, low-intensity signals. Here, we propose ReShuffle-MS, a region-guided data augmentation framework for MS data. Each spectrum is partitioned into a Main Discriminative Region (MDR) and a Peripheral Peak Region (PPR). By recombining signals within the PPR across samples of the same class while keeping the MDR intact, ReShuffle-MS generates structure-preserving augmented samples. On a clinical dataset for *Escherichia coli* (*E. coli*) levofloxacin resistance prediction, ReShuffle-MS delivered significant and consistent performance gains. It improved the average accuracy of classical machine learning models by 3.7% and enabled a one-dimensional convolutional neural network (CNN) to achieve 83.25% accuracy and 97.28% recall. Visualization using Grad-CAM revealed a shift from sparse, peak-dependent attention toward broader and more meaningful spectral patterns. Validation on the external DRIAMS-C dataset for ceftriaxone resistance further demonstrated that the method generalizes to a distinct laboratory setting and a different antibiotic target. These findings suggest that ReShuffle-MS can enhance the robustness and clinical utility of AI-based AMR prediction from routinely acquired MALDI-TOF spectra.

## 1. Introduction

Antimicrobial resistance (AMR) continues to escalate as a global public health threat, undermining the effectiveness of widely used antibiotics and complicating clinical decision-making during the early stages of infection management. When patients present with suspected bacterial infections, clinicians must frequently initiate empirical therapy before antimicrobial susceptibility testing (AST) results become available. This diagnostic gap, often lasting 24 to 48 h, creates substantial uncertainty and increases the likelihood of inappropriate therapy, either through unnecessary broad-spectrum coverage or inadequate treatment of resistant pathogens [1,2]. Clinical tools capable of providing early indications of resistance risk could therefore greatly enhance patient safety and antimicrobial stewardship.

Among bacterial pathogens, *Escherichia coli* (*E. coli*) remains one of the most common Gram-negative organisms encountered in clinical microbiology laboratories. According to the 2024 CHINET report, *E. coli* accounted for 18.2% of all clinical isolates in China, ranking first among reported species [3]. As a leading cause of urinary, abdominal, and bloodstream infections (BSI), *E. coli* can progress rapidly to sepsis or septic shock [4], underscoring the need for early and effective antimicrobial therapy. Given this clinical burden, fluoroquinolones—particularly levofloxacin—have long been widely used for urinary tract infections, intra-abdominal infections, and other *E. coli*–associated diseases owing to their broad-spectrum activity and convenient oral and intravenous formulations. However, the widespread use of levofloxacin has contributed to a continual rise in levofloxacin resistance among *E. coli* isolates, increasing the risk of treatment failure and adverse outcomes in severe infections and posing significant challenges to empirical therapy [5,6]. In response to these trends, the U.S. Food and Drug Administration (FDA) and other health authorities have issued guidelines discouraging empirical fluoroquinolone use unless susceptibility can be reasonably assured [7,8]. Consequently, methods capable of rapidly estimating resistance risk are becoming increasingly important, with direct implications for patient management and antimicrobial stewardship.

Although phenotypic AST remains the gold standard for determining bacterial resistance, matrix-assisted laser desorption ionization time-of-flight (MALDI-TOF) mass spectrometry (MS) enables rapid identification of pathogens by generating proteomic fingerprints of cultured isolates [9,10,11,12,13], allowing cost-effective, species-level assignment within minutes. This makes MALDI-TOF MS a promising method for AMR prediction using artificial intelligence (AI). However, extracting resistance-related information from MALDI-TOF MS spectra remains challenging. Resistance-associated proteomic variations are typically subtle, distributed across low-intensity regions, and frequently masked by the dominant ribosomal protein signals used for species identification [14], making them difficult to detect with conventional peak-based or manually engineered feature approaches. In *E. coli*, levofloxacin resistance is driven by *gyrA/parC* target-site mutations, plasmid-mediated *qnr* genes, and AcrAB–TolC efflux upregulation coupled with porin loss [15,16]. Because these determinants involve high-molecular-weight proteins or regulatory changes, they fall outside the mass range typically captured by routine MALDI-TOF MS, which predominantly detects 2–20 kDa ribosomal proteins. As a result, resistance does not manifest as discrete, identifiable peaks; instead, it is indirectly reflected through subtle, distributed spectral features across the mass range. Detecting such weak, global patterns requires modeling approaches capable of capturing diffuse and low-intensity signals rather than relying on isolated peak features [17,18]. These characteristics pose substantial analytical challenges and make levofloxacin-resistant *E. coli* a demanding yet informative benchmark for evaluating models designed to extract robust biological signals from noisy mass spectrometry data.

To overcome these limitations, recent studies have increasingly explored machine learning (ML) and deep learning (DL) models for AMR prediction from full mass spectra. Early ML approaches relied on handcrafted features, such as the presence of specific peaks, peak intensities, or peak areas [19,20,21]. However, reliance on a small set of selected peaks often results in substantial information loss and degraded predictive performance [22]. To preserve the complete spectral distribution and avoid premature feature selection, several studies have adopted full-spectrum modeling and applied DL architectures [23,24,25]. While deep neural networks are well suited for capturing nonlinear, high-dimensional relationships [26,27], their performance remains limited by the scarcity of labeled clinical spectra and the heightened risk of overfitting. Existing augmentation techniques, such as adding noise or applying simple intensity perturbations, have been used to expand the dataset [28] but do not account for the structured organization of real spectra and may distort biologically meaningful signals. As a result, there is a need for augmentation methods that avoid introducing external noise perturbations while enabling models to capture richer and more globally informative spectral patterns, thereby improving their robustness and generalization.

In this study, we present ReShuffle-MS, a region-guided spectral augmentation framework designed to improve the reliability of AMR prediction from MALDI-TOF MS and to provide earlier indications of potential resistance during the clinical decision-making window. ReShuffle-MS divides each mass spectrum into two functional zones: a Main Discriminative Region (MDR), which contains key informative features identified through importance analysis, and a Peripheral Peak Region (PPR), which contains distributed but individually weaker signals. The MDR is preserved to retain core biological information, while the PPR undergoes structured, intra-class recombination to simulate natural spectral variability without introducing noise-induced spectral distortions. This design encourages machine learning models to learn broad, stable spectral patterns rather than relying on isolated or spurious features. We evaluate ReShuffle-MS on a clinically relevant task: predicting levofloxacin resistance in *E. coli* isolates collected from Shanghai Sixth People’s Hospital. Across multiple classical machine learning classifiers, ReShuffle-MS consistently improves predictive performance, increasing average accuracy by 3.7 percentage points. When integrated with a convolutional neural network, the framework achieves an accuracy of 83.25% and a recall of 97.28%, underscoring its value in the early identification of potentially resistant isolates. Clinically interpretable metrics, including Very Major Error (VME) and Major Error (ME) rates, are also reported. In addition, we assess generalization on a public external dataset to evaluate robustness under differing laboratory conditions.

## 2. Materials and Methods

**Figure 1:**
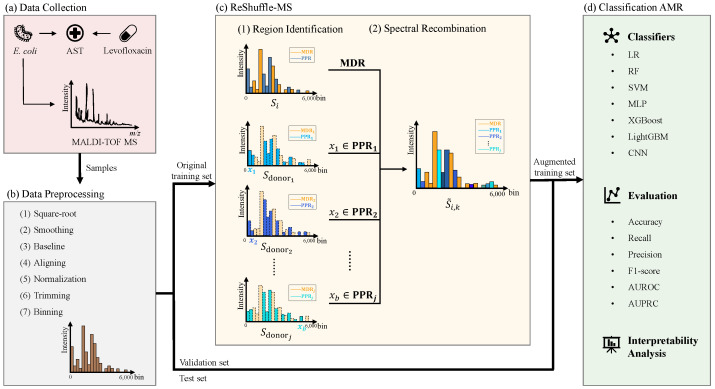
An overview of the workflow. (**a**) Data collection and selection: Clinical samples were collected, and those with incomplete metadata were excluded. (**b**) Data preprocessing: Spectra were standardized and binned into fixed-length feature vectors (6000 bins). (**c**) Data augmentation: The ReShuffle-MS augmentation strategy was applied to the training set, and the original and augmented spectra were merged to form the final training dataset. (**d**) Model training and evaluation: Models were trained using the augmented dataset. Performance was evaluated on the independent test set using metrics including accuracy, recall, precision, area under the receiver operating characteristic curve (AUROC), and area under the precision–recall curve (AUPRC).

This section details the experimental design and implementation of the study, following the workflow shown in Figure 1. First, clinical *E. coli* isolates were collected and profiled using MALDI-TOF MS (Figure 1a; Section 2.1). Raw spectra were then preprocessed to correct misalignments and standardized into fixed-length vectors (Figure 1b; Section 2.2). The dataset was split into training, validation, and test sets using a two-step stratified approach to avoid information leakage: 20% of the data were first isolated as a final, held-out test set using stratified sampling, and the remaining 80% were further divided into a training set (90%, or 72% of the total data) and a validation set (10%, or 8% of the total). Data augmentation was performed exclusively on the training set using the proposed ReShuffle-MS framework (Figure 1c; Section 2.3 and Section 2.4). Finally, classical machine learning and deep learning models were trained on the augmented data to predict resistance (Figure 1d; Section 2.5).

### 2.1. Data Sources

A total of 1045 clinical *E. coli* isolates were collected from patient specimens at Shanghai Sixth People’s Hospital between 25 March 2024, and 29 November 2024. Bacterial identification was performed using matrix-assisted laser desorption/ionization time-of-flight mass spectrometry (MALDI-TOF MS, Bruker, Bremen, Germany). All isolates were inoculated on trypticase soy agar with 5% sheep blood and incubated for 18–24 h at 35 °C, following standard clinical laboratory protocols. For MALDI-TOF MS analysis, a single colony was directly transferred to a MALDI target plate, covered with 70% formic acid, and then dried. A saturated solution of the MALDI matrix α-cyano-4-hydroxycinnamic acid (CHCA) was added to the sample. After drying, the samples were analyzed using the Microflex LT/SH mass spectrometer (Bruker Daltonik, Bremen, Germany) with the FlexControl software (version 3.1) and MALDI Biotyper (MBT) Compass version 4.1. Spectra were collected over a mass range of 2000–20,000 *m*/*z*. The antimicrobial susceptibility of *E. coli* to the antimicrobial agents was determined by Vitek Compact (bioMérieux, Marcy-l’Étoile, France) and BD Phoenix M50 (BD, Franklin Lakes, NJ, USA). Minimum inhibitory concentrations (MICs) were interpreted according to the Clinical and Laboratory Standards Institute (CLSI) 2024 guidelines [29]. For levofloxacin, isolates were categorized as susceptible (MIC ≤ 2 mg/L), intermediate (MIC = 4 mg/L), or resistant (MIC ≥ 8 mg/L).

The AST results were converted into binary labels for subsequent machine learning analysis: isolates categorized as intermediate (I) or resistant (R) were assigned to Class 1 (resistant), while susceptible (S) isolates were labeled as Class 0 (susceptible). This classification follows the CLSI guidelines [29] and reflects clinical practice, as intermediate susceptibility indicates an uncertain therapeutic outcome and is therefore typically managed as resistance, particularly in severe infections where empirical therapy must be applied with caution. The final dataset comprised 310 isolates (29.67%) classified as Class 0 and 735 isolates (70.33%) classified as Class 1.

### 2.2. Data Preprocessing

All MALDI-TOF MS spectra underwent a standardized preprocessing pipeline to ensure data quality and comparability. The preprocessing followed these seven steps:

1. Square-root transformation was applied to the raw intensity to stabilize variance.
2. Smoothing was performed using the Savitzky–Golay filter [30] with a window length of 21 and a polynomial order of 3.
3. Baseline correction was achieved by removing baseline estimates fitted using the Asymmetric Least Squares (ALS) [31] algorithm.
4. Calibration and spectral alignment were performed using the reference protein masses listed in Table 1 (reported in Daltons, Da, with a tolerance of ±300 ppm). During calibration, the instrument aligns the measured peak positions with the theoretical *m*/*z* values derived from these known molecular masses. Spectral alignment was then applied to correct residual *m*/*z* shifts across samples, ensuring consistent peak positioning for downstream analysis.
5. Total ion current (TIC) normalization was applied to each spectrum.
6. Spectra were trimmed to include *m*/*z* values within the 2000–20,000 Da range.
7. The trimmed spectra were binned into intervals of fixed width (3 Da) along the *m*/*z* axis. For each bin, the total intensity was calculated by summing all signal values within the interval. Thus, each sample was represented as a 6000-dimensional feature vector, corresponding to the number of bins across the specified *m*/*z* range.

After preprocessing, the dataset was randomly divided into three mutually exclusive subsets using stratified sampling to preserve the class balance between susceptible (S) and resistant (R) isolates: a training set (n=752; 72%), a validation set (n=84; 8%), and an independent test set (n=209; 20%). The partitioning was performed using a fixed random seed (random_state = 0) to ensure reproducibility. Stratified sampling ensured the original class proportions of resistant (Class 1: R) and susceptible (Class 0: S) cases were maintained across the training, validation, and test subsets. Specifically, the distribution of isolates within these subsets comprised: training (529 R, 223 S), validation (59 R, 25 S), and test (147 R, 62 S). We utilized this internal data-splitting strategy to provide a rigorous within-antibiotic evaluation of the framework. There is no spectrum shared between subsets to maintain strict data independence at any stage of model development or evaluation. Therefore, this design approximates the robustness of an external validation. All datasets are available in the Zenodo repository at https://doi.org/10.5281/zenodo.17139697.

### 2.3. Region Identification

The full-length mass spectrum was partitioned into two complementary subregions: a MDR and a PPR. The MDR was guided by feature importance scores derived from a Random Forest (RF) classifier [32]. Here, the RF was trained as a binary classifier to discriminate susceptible (S, class 0) and resistant (R, class 1) isolates based on the training labels, and feature importance was computed with respect to this binary task. RF is an ensemble learning method that constructs a set of decision trees, denoted as $T=\{T_{1},T_{2},\ldots ,T_{H}\}$, each trained on a bootstrapped subset of the training data.

For each feature $x_{b}$, its importance is defined as the average reduction in Gini impurity across all trees in the RF where $x_{b}$ is used as a splitting variable. The importance is computed as:

(1) $$\mathrm{Importance}\left(x_{b}\right)=\frac{1}{H}\sum_{h=1}^{H}\sum_{t\in T_{h}}1_{\mathrm{split}\;\mathrm{on}\;x_{b}}\cdot \Delta \mathrm{Gini}\left(t\right)$$

where *H* is the number of trees in the RF, ΔGini(t) represents the reduction in Gini impurity at node *t* in tree $T_{h}$, and $1_{\mathrm{split}\;\mathrm{on}\;x_{b}}$ is an indicator function that equals 1 if feature $x_{b}$ is used for splitting at node *t*, and 0 otherwise.

The Gini impurity at node *t* is calculated as:

(2) $$\mathrm{Gini}\left(t\right)=\sum_{c=1}^{C}{\hat{p}}_{tc}\left(1-{\hat{p}}_{tc}\right)$$

where ${\hat{p}}_{tc}$ is the estimated probability of samples at node *t* belonging to class *c*. This metric reflects the probability of incorrect classification if a sample is randomly labeled according to the class distribution at that node.

Features exceeding a predefined threshold were selected for the MDR. Specifically, a bin *i* was included in the MDR if its importance satisfied:

(3) $${\mathrm{Importance}}_{i}\geq \alpha \cdot \bar{\mathrm{Importance}}$$

where $\bar{\mathrm{Importance}}$ is the mean importance across all features, and α is a tunable threshold parameter. The details behind this regional division strategy can be found in Supplementary Figure S1, where the mean spectra of resistant and susceptible isolates show strong visual similarity, and the importance profile identifies discriminative bins. In this study, α was set to 2.15 according to the benchmarking results illustrated in Supplementary Figure S2A.

Each bin selected based on feature importance was further expanded to account for local dependencies and correlations with neighboring peaks, by including adjacent bins within a symmetric window of ±N positions along the *m*/*z* axis. The resulting set of bins collectively defined the final MDR. In this study, *N* was set to 1 to ensure the formation of biologically coherent spectral regions, as shown in Supplementary Figure S2B. All remaining bins not included in the MDR were designated as the PPR and used for subsequent intra-class recombination.

### 2.4. Spectral Recombination

Following the identification of the MDR and PPR, the spectral recombination process was applied. The key idea is to selectively recombine intensity in the PPR region from same-class donor samples, while preserving the MDR unchanged.

Let $D=\{S_{1},S_{2},\ldots ,S_{n}\}$ be the original spectra in the training set. For each spectrum $S_{i}$, we generated *k* augmented spectra ${\tilde{S}}_{i,k}$ by combining bins from same-class samples. The intensity value at each bin *b* is defined as:

(4) $${\tilde{S}}_{i,k}\left[b\right]=\left\{\begin{array}{cc} S_{i}\left[b\right], & \mathrm{if}\;b\in \mathrm{MDR}\\ S_{{\mathrm{donor}}_{j,k}}\left[b\right], & \mathrm{if}\;b\in \mathrm{PPR}\;\mathrm{and}\;\mathrm{Class}\left(S_{{\mathrm{donor}}_{j,k}}\right)=\mathrm{Class}\left(S_{i}\right) \end{array}\right.$$

Here, the MDR bins in each augmented spectrum are directly retained from the original spectrum $S_{i}$, while PPR bins are independently drawn from random donor spectra, denoted as $S_{\mathrm{donor}}$, within the same class. This process is repeated *k* times for each original sample $S_{i}$ to create an augmented dataset. For each bin and each augmentation instance *k*, the donor spectrum is sampled independently, ensuring that every ${\tilde{S}}_{i,k}$ encodes a unique recombination of intra-class spectral patterns. This randomness does not create new structural information. Instead, it helps disrupt incidental or spurious peaks, thereby encouraging the model to focus on more robust and essential structural features that remain consistent across perturbations. In this study, the augmentation factor was set to k=6, based on preliminary experiments that balanced performance gains and computational cost (Supplementary Figure S2C). The final augmented training set was formed by merging all recombined spectra with the original training data, resulting in a total of 5264 samples (1338 susceptible, 3174 resistant), while maintaining the original prevalence (≈30% vs. 70%). This natural imbalance was intentionally preserved, as it reflects the true clinical distribution of resistant isolates and supports realistic model calibration.

### 2.5. Model Training and Evaluation

Seven classifiers were used to assess the broad applicability of ReShuffle-MS. These included six classical machine learning algorithms: Logistic Regression (LR), RF, Support Vector Machine (SVM), Multi-layer Perceptron (MLP), XGBoost, and LightGBM. In addition, one deep learning model was employed: MSDeepAMR, a convolutional neural network (CNN)-based architecture. These models were selected based on their proven effectiveness and frequent use in previous MALDI-TOF-based AMR prediction studies [22,23,33,34,35,36,37,38]. XGBoost (v2.1.4) and LightGBM (v4.6.0) were incorporated via their official packages and interfaced through scikit-learn-compatible APIs. Other classical models were implemented using the scikit-learn library (v1.0.2) [39] in Python 3.9.21. Each classifier’s hyperparameters were independently optimized via grid search on the validation set. The search space included parameters such as regularization strength, number of estimators, learning rates, and hidden layer sizes, depending on the model architecture. Hyperparameters were tuned independently for Baseline (original training data) and ReShuffle-MS (augmented training data), with identical search spaces. The results were finally reported on the same held-out test set. A detailed summary of all hyperparameter ranges is provided in Supplementary Table S1. MSDeepAMR, a representative deep learning model [27,38], was used to assess the effectiveness of the proposed ReShuffle-MS framework. The original MSDeepAMR architecture was retained, with only minimal modifications (e.g., learning rate, early stopping) applied to adapt it to our dataset.

All models were trained on the augmented training dataset, with the validation set used for hyperparameter tuning and early stopping. The test set remained completely unseen throughout data augmentation and model tuning, serving as an independent benchmark for final performance evaluation. Evaluation metrics included accuracy, recall, precision, F1-score, AUROC, and AUPRC. Recall was emphasized due to its clinical importance in reducing the likelihood of false negatives and the risk of missed resistant cases, which could negatively impact patient outcomes. To ensure robustness and statistical reliability, all reported results represent the mean of five independent training runs.

## 3. Results

This section presents the validation of the ReShuffle-MS framework, conducted on an in-house *E. coli* dataset and an external public dataset. We begin by demonstrating performance improvements over the baseline across seven classifiers (Section 3.1). Subsequently, the contributions of the framework’s key components are evaluated through a series of ablation studies (Section 3.2, Section 3.3 and Section 3.4), followed by an examination of the effect of the augmentation factor *k* on model performance (Section 3.5). The framework’s cross-dataset generalizability is then assessed using the public DRIAMS-C [23] dataset (Section 3.6). Finally, an interpretability analysis provides insights into the model’s altered learning behavior (Section 3.7).

### 3.1. The Effectiveness of ReShuffle-MS Across Classifiers

To evaluate the effectiveness of the proposed ReShuffle-MS framework, we first established baseline classification performance using both traditional machine learning classifiers and a CNN (MSDeepAMR architecture) on the original, non-augmented MALDI-TOF MS dataset for *E. coli* resistance prediction.

Applying ReShuffle-MS with its optimized parameters (α=2.15, N=1, k=6; see Supplementary Figure S2) led to consistent performance improvements across all classifiers, especially the CNN model. As shown in Table 2, the CNN’s accuracy increased from 73.21% to 83.25%, and recall from 76.87% to 97.28%. This significant reduction in false negatives is an essential consideration in clinical diagnostics where missed resistant cases can lead to inappropriate antibiotic use. The F1-score improved to 0.8910, and AUROC reached 0.8391.

The benefits of ReShuffle-MS were not limited to deep learning models. All six classical machine learning classifiers (LR, RF, SVM, MLP, XGBoost, and LightGBM) showed consistent improvements in both accuracy and AUROC. On average, ReShuffle-MS led to a 3.7 percentage point gain in accuracy across these models. This consistency supports the generalizability of ReShuffle-MS to a variety of learning paradigms.

To further relate these quantitative improvements to their biological and clinical implications, we calculated the VME and ME rates based on the confusion matrix of the ReShuffle-MS CNN model on the independent test set. False negatives (resistant isolates predicted as susceptible) correspond to VME, while false positives (susceptible isolates predicted as resistant) correspond to ME.

For the best-performing CNN model on the independent test set (n=209 isolates; 62 susceptible and 147 resistant/intermediate isolates), the confusion-matrix outcomes were: true positives (TP) = 143, false negatives (FN) = 4, false positives (FP) = 31, and true negatives (TN) = 31. According to standard AST error definitions, the corresponding rates were:

VME rate (FN/Total R) =4/147=2.72%

ME rate (FP/Total S) =31/62=50.00%

In AST evaluation, VME is commonly regarded as a critical safety-related metric, as it reflects the failure to identify resistant isolates. In this test setting, the observed VME rate of 2.72% indicates that the model correctly identified the majority of resistant isolates. At the same time, the high sensitivity (Recall ≈97.28%), together with the accompanying ME rate (50.00%), reflects a sensitivity-oriented performance profile, achieved at the cost of an increased false-positive rate.

### 3.2. The Main Discriminative Region Drives Predictive Performance

We first validate whether the RF-derived region partition (MDR vs. PPR) meaningfully reflects differential discriminative contributions to AMR prediction under non-augmented training. To define the MDR in this study, we applied a feature importance threshold (α=2.15) based on the Gini index from a RF model, and expanded each selected bin by one position (N=1) on both sides to form contiguous regions. A detailed exploration of the selection of α and *N* is provided in Supplementary Figure S2.

We trained the MSDeepAMR model under three distinct, non-augmented training settings:

1. Original-Full: using the full, original spectra.
2. Original-MDR: using only the MDR bins.
3. Original-PPR: using only the PPR bins.

As shown in Table 3, the model trained on only the PPR performed poorly, with an accuracy of 66.03% and an AUROC of 0.5899, confirming that the PPR lacks substantial independent predictive power, although it still retains some classification ability and may contain complementary discriminative information. In stark contrast, the model trained on only the MDR not only performed well but also slightly outperformed the full-spectrum baseline in key metrics, including recall and F1-score. These results support the validity of the RF-derived partition: the MDR concentrates the primary discriminative signal, whereas the PPR alone is insufficient for accurate prediction but may contain complementary information when combined with the MDR.

### 3.3. Structured Recombination Outperforms Generic Augmentation

We compared the performance of our framework against two common augmentation strategies: Gaussian noise and TIC disturbance. For a fair comparison, these generic augmentations were applied to the PPR only, matching the application scope of our method. Meanwhile, an ablation study investigating the effect of augmentation on the MDR is carried out in Section 3.4.

1. ReShuffle-MS: the data augmentation method we proposed.
2. Gaussian Noise Injection: adds Gaussian noise to PPR bins, where the noise standard deviation is set to 5% of the original intensity.
3. TIC Disturbance: applies random multiplicative scaling to PPR intensities to modulate the TIC.

All three augmentation strategies were implemented with identical augmentation factors and applied to the same original dataset. For comparison, we also report the performance of the non-augmented baseline model. Results are summarized in Table 4. ReShuffle-MS outperformed both generic strategies. In contrast, the simpler noise-based methods even offered detrimental effects. Gaussian Noise reduced accuracy to 72.25%, despite a high precision (86.18%) possibly due to under-detection of resistant cases, the resulting F1-score (0.7852) was lower than that of the non-augmented baseline (0.8014), indicating a decline in balanced performance. TIC Disturbance offered some gains in recall and F1-score but remained clearly inferior to ReShuffle-MS.

These findings provide evidence that the benefit of our framework is not merely due to an increase in data volume, but is dependent on the augmentation’s structural integrity. Conversely, unstructured perturbations, even when applied only to peripheral regions, appear to confuse the classifier rather than enhancing its classification ability.

### 3.4. Region-Guided and Structurally-Aware Designs Are Key to Performance

To determine how spectral recombination should be designed to improve learning while preserving discriminative structure, we evaluated a set of controlled recombination strategies that differ in region preservation and structural constraints. Here, the previously validated regional partition is treated as a fixed structural prior, and the focus is placed exclusively on how different recombination designs interact with this structure, thereby revealing which constraints are necessary for effective augmentation. Specifically, we examined two core design principles of ReShuffle-MS: (1) preserving the MDR, and (2) enforcing class-consistent and position-consistent recombination within the PPR. Four augmentation strategies were compared:

1. Shuffle-All: a naive recombination baseline that shuffles all bins, including those in the MDR.
2. ReShuffle-MS: the proposed method, in which the MDR is fixed and recombination is applied only within the PPR.
3. Shuffle-MDR-only: a reversal of our approach that shuffles only the MDR, leaving the PPR unchanged.
4. MDR Removal: a strategy in which the MDR is completely removed prior to training, testing whether the augmented PPR alone suffices for classification.

As shown in Table 5, ReShuffle-MS consistently outperformed all other strategies. Shuffling the MDR led to notable performance degradation, confirming that disrupting discriminative structures in the MDR is harmful. Performance dropped most sharply in the MDR Removal condition (Accuracy: 68.42%, AUROC: 0.6178), providing direct evidence that the PPR, despite augmentation, cannot compensate for the absence of the MDR. These results indicate that preserving the MDR during augmentation is necessary.

Next, we evaluated how recombination should be performed to maximize its benefit. Specifically, we investigated two structural constraints: (i) whether bin values are recombined only among samples from the same class (intra-class) versus any sample (cross-class), and (ii) whether recombination is restricted to the same bin position (positional correspondence) versus any position (free-bin). These two dimensions yielded a 2 × 2 ablation matrix, summarized in Table 6.

The results in Table 7 demonstrate that both structural constraints are crucial. Removing label consistency (Global Shuffle) resulted in a substantial decrease in accuracy and F1-score, likely due to contradictory information introduced by mixing features across different resistance classes. Similarly, removing positional correspondence (Free-Bin Shuffle) led to lower accuracy and AUROC, suggesting that preserving spatial context along the *m*/*z* axis is essential for maintaining discriminative integrity during augmentation.

### 3.5. Optimization of Augmentation Factor *k*

A primary motivation for data augmentation is to enhance model performance, particularly when training data is scarce. To validate this role of ReShuffle-MS, we conducted experiments under two simulated data-limited scenarios by using reduced training sets of n=360 and n=500. For each scenario, we evaluated the CNN (MSDeepAMR) model’s performance with no augmentation (k=0), moderate augmentation (k=5), and extensive augmentation (k=10).

The results, summarized in Table 8, reveal two key insights. First, ReShuffle-MS demonstrated its principal utility in the most data-limited setting (n=360), where accuracy dramatically increased from 50.00% to 64.00% as *k* was raised to 10. Similarly, in the setting with 500 training samples, model accuracy increased from 70.33% (k=0) to 75.60% (k=5), while recall improved from 77.55% to 82.99%. This confirms the framework’s effectiveness at compensating for data scarcity. Second, the results underscore the irreplaceable value of authentic data: larger training sets even without augmentation yielded better performance compared to smaller but augmented datasets, reaffirming the importance of data quantity in MS-based AMR prediction. Furthermore, a performance plateau was observed in the 500-sample setting, with no significant accuracy gain between k=5 and k=10. This saturation effect suggests that the benefits of augmentation are not infinite. This is because data augmentation does not create new biological information but merely helps the model learn the existing patterns in the input data more comprehensively. Consequently, once the augmented data has adequately covered the underlying distribution of the limited dataset, further augmentation provides diminishing returns. This highlights the importance of selecting an optimal *k* to balance performance gains and computational cost.

### 3.6. Performance on Public DRIAMS Dataset

To assess generalizability and effectiveness across datasets and antibiotic targets, the ReShuffle-MS framework was evaluated on the publicly available DRIAMS-C dataset, which comprises 913 MALDI-TOF spectra of *E. coli* collected at the Cantonal Hospital of Aarau, Switzerland [23]. As resistance labels for levofloxacin were not available in DRIAMS-C, we instead focused on predicting ceftriaxone resistance. This task was also included as a benchmark in the original DRIAMS study [23], allowing for direct comparison under consistent experimental conditions. The dataset was split into 657 training samples (551 S, 106 R), 73 validation samples (61 S, 12 R), and 183 test samples (153 S, 30 R), revealing a substantial class imbalance (∼16% R).

As summarized in Table 9, the model trained using ReShuffle-MS achieved an accuracy of 84.15%, an AUROC of 0.8584, and an AUPRC of 0.5356. For comparison, Weis et al. [23] reported an AUROC of 0.66 for the same ceftriaxone-resistance task on the DRIAMS-C subset, and a slightly higher AUROC (0.74±0.02) and AUPRC (0.30±0.03) only when trained on the much larger DRIAMS-A dataset. Despite being trained solely on the smaller DRIAMS-C dataset without any external data, our framework achieved superior performance, indicating that ReShuffle-MS enables more efficient learning even under data-limited and imbalanced conditions. Despite the imbalance, ReShuffle-MS substantially outperformed the logistic regression baseline reported by Weis et al. [23] (AUROC = 0.66), highlighting its improved generalization capability on external data.

These results highlight the cross-antibiotic and cross-platform versatility of ReShuffle-MS. By enhancing spectral signal utilization through region-guided recombination, the framework maintained its discriminative power even when applied to a novel antibiotic phenotype (ceftriaxone) with different underlying resistance signatures. This external validation provides evidence that ReShuffle-MS functions as an antibiotic-agnostic data augmentation framework, underscoring its potential for broad clinical application in MALDI-TOF–based antimicrobial resistance prediction.

### 3.7. Interpretability Analysis Reveals a Shift in Model Attention and Learning Behavior

We applied Gradient-weighted Class Activation Mapping (Grad-CAM) [40] to visualize how ReShuffle-MS influences the internal learning behavior of the CNN. The resulting one-dimensional attention maps indicate the relative contribution of individual spectral bins to the model’s predictions, providing insight into how discriminative information is utilized during decision-making. Given the class imbalance in the dataset and the sensitivity-oriented training objective, improvements in resistance prediction performance alone may not fully capture how the model utilizes spectral information. Therefore, Grad-CAM analysis is employed primarily to examine changes in attention distribution and decision strategy.

**Figure 2:**
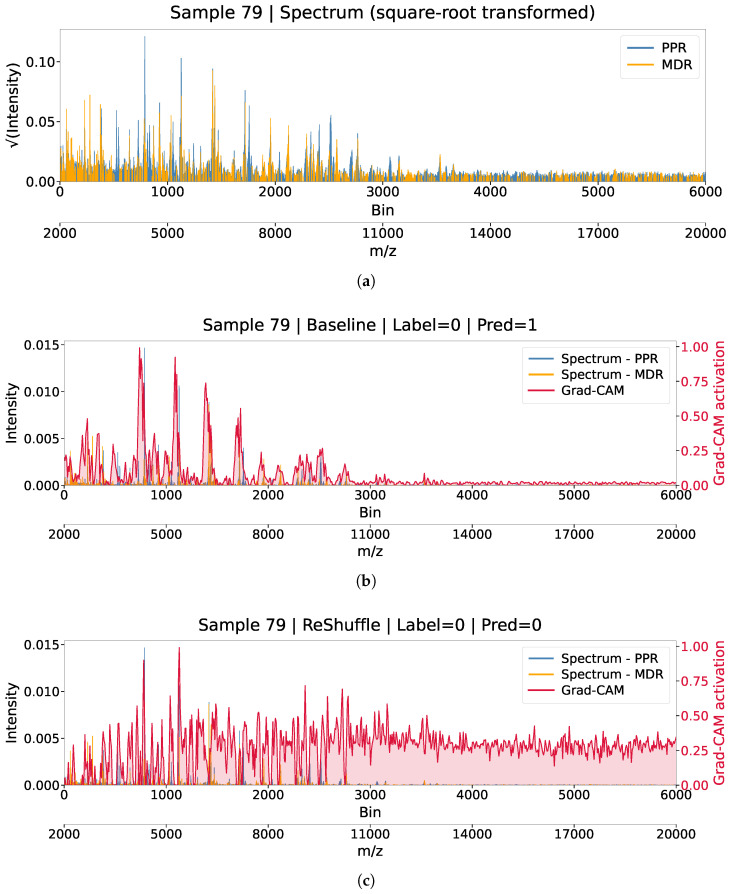
Grad-CAM case study of Sample 79, a false positive corrected by ReShuffle-MS. (**a**) Square-root transformed spectrum of Sample 79 (S isolate) with MDR (orange) and PPR (blue). (**b**) Baseline model misclassifies the sample as resistant, showing sparse peak-driven attention. (**c**) ReShuffle-MS correctly classifies the sample; the attention map is denser and more broadly distributed.

To characterize the behavioral shift introduced by ReShuffle-MS, we first analyzed individual case studies where the framework corrected classification errors made by the baseline model. We first focus on a representative false-positive case (FP → TN), which provides a more conservative test of whether ReShuffle-MS can mitigate over-reliance on isolated, high-intensity peaks and reduce spurious resistance predictions, because correcting false positives requires suppressing spurious resistance signals rather than benefiting from a general tendency toward the resistant class. Figure 2 presents such an example: Sample 79, a susceptible isolate misclassified as resistant by the baseline model (FP). As shown in Figure 2b, the baseline model’s Grad-CAM attention is sharply concentrated within a narrow *m*/*z* range, focusing on a few isolated bins corresponding to high-intensity peaks. This sparse and localized activation pattern suggests that the model may have relied on a small number of prominent signals as shortcut cues for classification. In contrast, the ReShuffle-MS model correctly identifies the sample as susceptible (TN) and exhibits a broader, more evenly distributed attention pattern across the spectrum (Figure 2c). This reflects a shift in the model’s decision strategy: from peak-centric heuristics to a more holistic, pattern-based interpretation.

Similar attention redistribution was observed in additional cases, including FN → TP and TP → TP examples (Supplementary Figure S3), indicating that the observed change in attention behavior is not restricted to a single error type.

**Figure 3:**
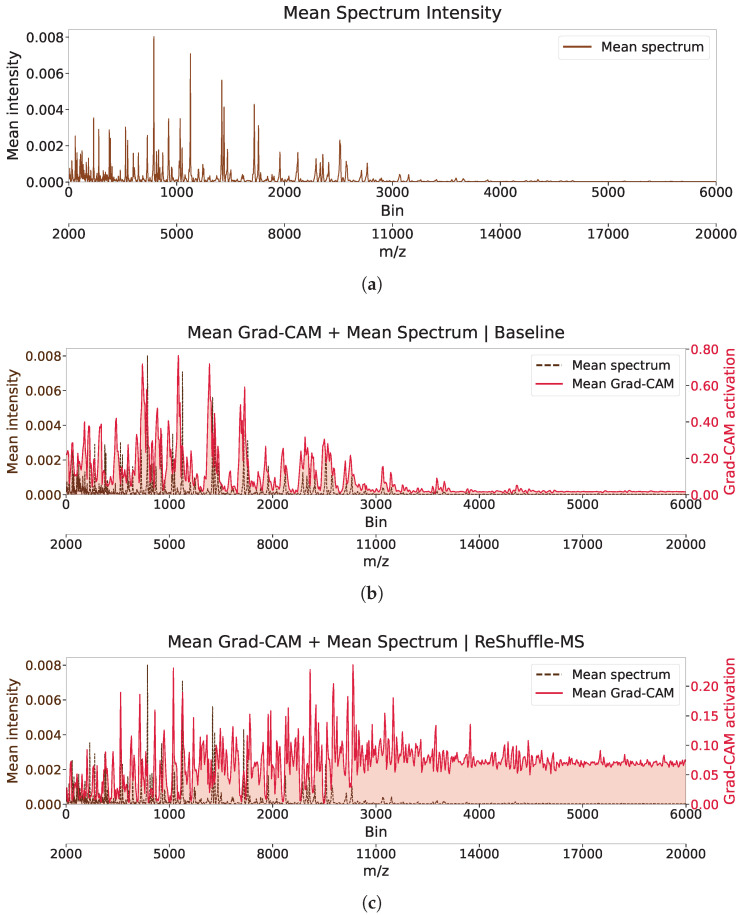
Average Grad-CAM attention across the entire test set. (**a**) Mean spectrum of test samples. (**b**) Average attention map of the baseline model, showing strong correlation with spectral intensity and reliance on high-intensity peaks. (**c**) Average attention map of ReShuffle-MS, demonstrating broader and more evenly distributed attention across the spectrum, including low-intensity regions.

To assess whether this behavioral shift generalizes across the dataset, we computed the average Grad-CAM attention maps over all test samples (Figure 3). The baseline model’s average attention closely mirrors the average spectral intensity (Figure 3b), indicating a strong bias toward high-intensity peaks and a tendency to conflate signal strength with discriminative importance. Conversely, the ReShuffle-MS model produces a more broadly distributed attention profile (Figure 3c), demonstrating increased sensitivity to low-intensity regions, thereby capturing more subtle and distributed resistance-related features. These observations suggest that ReShuffle-MS promotes a change in learning behavior, encouraging the model to rely on more distributed spectral information rather than a limited set of dominant peaks, thereby supporting more stable and reliable decision-making.

## 4. Discussion

In this study, we developed ReShuffle-MS, a region-guided and structure-aware data augmentation framework that aims to improve AMR prediction from MALDI-TOF mass spectrometry data when using machine-learning models under limited sample availability. The framework consistently improved predictive performance across multiple classifiers, indicating that structured spectral recombination can support more stable feature learning and partially mitigate the impact of data scarcity.

Traditional MALDI-TOF–based AMR prediction approaches typically rely either on handcrafted peak selection or on biologically motivated filtering rules [41,42,43], which may remove subtle but informative spectral cues important for classification [44,45,46,47]. In contrast to these approaches, ReShuffle-MS is formulated as a structure-aware yet data-driven augmentation framework that retains the full spectrum and separates it into a MDR and a PPR. In the present study, the MDR is instantiated using feature importance scores derived from a RF model [32]. This choice is not intrinsic to the ReShuffle-MS framework, but rather represents an objective and reproducible mechanism that avoids subjective peak selection. Although the current study adopts a fully data-driven region definition and does not rely on explicit biological markers, the region-based formulation of ReShuffle-MS is intentionally designed as a flexible and extensible abstraction of discriminative structure. This design allows biological prior knowledge, such as established resistance markers or biological prior information, to be incorporated in future work when available, without constraining the framework to any predefined biological assumptions. The PPR is used for controlled intra-class recombination, introducing structured variability that encourages models to rely on stable and recurrent patterns rather than isolated, sample-specific events. Importantly, this augmentation strategy does not introduce external noise or artificial discriminative structures, thereby minimizing the risk of contaminating biologically meaningful spectral information. Independent species-level validation supporting this design choice is provided in the Supplementary Material (Supplementary Table S2 and Figure S4).

From a biological perspective, the learning behavior promoted by ReShuffle-MS aligns with the intrinsic characteristics of MALDI-TOF spectra. MALDI-TOF instruments primarily capture ribosomal proteins within the 2–20 kDa range and therefore do not directly detect high-molecular-weight resistance markers [48,49], such as β-lactamases (∼30 kDa) [50] or porins like OmpC (∼40 kDa) [51]. As a result, artificial intelligence models must infer resistance indirectly through low-mass proteomic footprints. Grad-CAM visualization in our analysis showed that models trained with ReShuffle-MS distribute attention across a wider range of subtle spectral features instead of focusing narrowly on a small number of high-intensity peaks. This shift reflects a change in learning behavior toward more distributed, pattern-based representations, which may be more compatible with the indirect and low-mass proteomic signals captured by MALDI-TOF spectra. At the same time, these results should be interpreted in the context of the dataset characteristics and training objectives. The training data exhibit class imbalance, with resistant isolates being more prevalent than susceptible ones. Such imbalance can influence the learned decision boundary and increase the overall tendency of the model to predict the majority (resistant) class, thereby contributing to the observed trade-off between low VME and elevated ME rates. Importantly, such effects primarily affect prediction frequency rather than the spatial distribution of attention across the spectrum. Therefore, while class imbalance may partly explain changes in classification outcomes, it does not fully account for the systematic redistribution of Grad-CAM attention from a small number of dominant peaks toward broader, low-intensity regions. In this sense, the Grad-CAM analysis is intended to characterize changes in learning behavior induced by ReShuffle-MS, independent of class prevalence or decision threshold effects.

ReShuffle-MS also addresses two persistent challenges in MS-based AMR prediction: the dependence on high-quality, large-scale datasets [52,53,54] and the susceptibility to batch effects [55,56,57]. Importantly, the observed performance improvements cannot be explained solely by an increase in training sample size or by generic perturbation effects. As explored in the ablation analyses (Section 3.3), commonly used augmentation strategies—such as adding random noise or performing global intensity shuffling—do not explicitly account for the regional organization of MALDI-TOF spectra and may alter spectral structure in ways that are difficult to interpret biologically. In contrast, ReShuffle-MS is formulated to respect the inherent regional structure of the spectrum by preserving the MDR while applying controlled, class-consistent recombination within the PPR. Rather than introducing external noise, this design aims to reuse existing spectral information in a structured manner, thereby exploring plausible within-class variability. To assess whether this structure-aware design remains effective in the presence of batch effects and inter-laboratory variability, we evaluated the framework on the external DRIAMS-C dataset, which combines limited sample size, pronounced class imbalance, and laboratory-specific acquisition conditions. In this setting, ReShuffle-MS achieved high accuracy for ceftriaxone resistance prediction, and the AUROC showed a marked improvement over baselines. These results indicate that the method is not tied to any specific drug mechanism and can be readily adapted to different pathogen–antibiotic combinations, even under data-limited conditions, without modifying the underlying model architecture. Although broader validation is needed across additional bacterial species, antibiotic classes, and MS instrumentation platforms [58], this external experiment provides additional evidence of its potential.

From a clinical perspective, machine-learning models derived from MALDI-TOF spectra are not intended to replace phenotypic AST. Rather, within the scope of the present study, ReShuffle-MS should be viewed as a proof-of-concept framework that supports early screening or triage during the 24–48-h interval between species identification and AST confirmation. In this setting, model predictions are used to provide an earlier risk signal rather than a definitive therapeutic decision. FP predictions (i.e., susceptible isolates predicted as resistant) may lead to more cautious empirical treatment or prioritization of confirmatory testing, and can be corrected once standard AST results become available. Conversely, FN predictions (i.e., resistant isolates predicted as susceptible) carry the risk of delayed recognition of resistance and prolonged exposure to suboptimal therapy, which may adversely affect patient outcomes. Framing ReShuffle-MS as an early-warning or triage aid helps contextualize this trade-off: the model is designed to complement, rather than replace, routine AST by accelerating risk assessment and supporting timely clinical decision-making.

Overall, ReShuffle-MS introduces a structure-aware spectral augmentation strategy that emphasizes robustness, interpretability, and cross-dataset generalization under data-limited conditions. By leveraging information already present in routinely acquired MALDI-TOF spectra and reshaping model learning behavior toward distributed spectral patterns, the framework offers a promising approach for improving the reliability of AI-based AMR prediction models.

## 5. Conclusions

The ReShuffle-MS framework proposed in this study demonstrates that region-guided data augmentation can effectively strengthen machine-learning models for antimicrobial resistance prediction from MALDI-TOF mass spectrometry data. By preserving the integrity of the MDR while applying structure-aware recombination within the PPR, the method reduces reliance on isolated peaks and promotes the learning of more stable and informative spectral patterns. This design contributes to improved model robustness, particularly under data-limited conditions.

Across multiple classifiers, ReShuffle-MS consistently enhanced predictive performance on the in-house dataset, supporting its effectiveness as a structure-aware augmentation strategy for MALDI-TOF–based resistance prediction. Evaluation on the external DRIAMS-C dataset further demonstrated that the method generalizes across laboratory settings and antibiotic targets, indicating its potential applicability to a broader range of pathogen–antibiotic combinations.

Taken together, these findings suggest that ReShuffle-MS represents a practical direction for advancing AI-assisted resistance prediction from routinely collected MALDI-TOF spectra. Further work, including prospective multi-center studies and expansion to additional species and antibiotics, is warranted to fully assess its generalizability.

## Figures and Tables

**Table 1:** Mass error range of calibration points (±300 ppm).

No.	Reference Protein	Mass (Average Mass, Da) *	±300 ppm Tolerance Range (Da)
1	RL29 [M+2H]^2+^	3637.8	3636.7–3638.8
2	RS32 [M+H]^+^	5096.8	5095.3–5098.3
3	RS34 [M+H]^+^	5381.4	5379.8–5383.0
4	RS33meth [M+H]^+^	6255.4	6253.5–6257.3
5	RL29 [M+H]^+^	7274.5	7272.3–7276.7
6	RS19 [M+H]^+^	10,300.1	10,297.0–10,303.2
7	RNase A [M+H]^+^	13,683.2	13,679.1–13,687.3
8	Myoglobin [M+H]^+^	16,952.3	16,947.2–16,957.4

* Reference protein masses are based on the Bruker manufacturer specifications.

**Table 2:** Performance comparison of seven classifiers with and without ReShuffle-MS on the MALDI-TOF dataset. All models were evaluated on the same held-out test set. Bold values indicate the better result for each metric.

Model	Augmentation	Accuracy (%)	Recall (%)	Precision (%)	F1-Score	AUROC
CNN (MSDeepAMR)	Baseline	73.21	76.87	**83.70**	0.8014	0.7897
	ReShuffle-MS	**83.25**	**97.28**	82.18	**0.8910**	**0.8391**
LR	Baseline	74.64	**84.35**	80.52	0.8239	0.7507
	ReShuffle-MS	**77.03**	83.67	**83.67**	**0.8367**	**0.7603**
RF	Baseline	75.12	**97.28**	74.87	0.8462	0.8287
	ReShuffle-MS	**78.47**	96.60	**78.02**	**0.8632**	**0.8389**
SVM	Baseline	71.29	**94.56**	72.77	0.8225	0.7458
	ReShuffle-MS	**77.03**	86.38	**81.94**	**0.8411**	**0.7847**
MLP	Baseline	74.64	85.03	80.13	0.8251	0.7742
	ReShuffle-MS	**76.08**	**85.03**	**81.70**	**0.8333**	**0.7677**
XGBoost	Baseline	74.64	87.07	79.01	0.8285	0.7797
	ReShuffle-MS	**80.86**	**91.16**	**83.23**	**0.8701**	**0.8272**
LightGBM	Baseline	76.56	90.48	79.17	0.8444	0.8163
	ReShuffle-MS	**80.38**	**93.88**	**81.18**	**0.8707**	**0.8373**

**Table 3:** Performance of CNN models trained on different spectral regions of the original (non-augmented) mass spectra. Bold values indicate the best result for each metric.

Strategy	Data Augmentation	Accuracy (%)	Recall (%)	Precision (%)	F1-Score	AUROC
Original-Full	k=0	73.21	76.87	**83.70**	0.8014	**0.7897**
Original-MDR	k=0	**75.12**	**87.76**	79.14	**0.8323**	0.7487
Original-PPR	k=0	66.03	87.76	70.88	0.7842	0.5899

**Table 4:** CNN performance under baseline (no augmentation) and three augmentation strategies targeting the PPR: ReShuffle-MS, Gaussian noise, and TIC disturbance. Metrics evaluated on a fixed test set; bold indicates the best.

Strategy	Accuracy (%)	Recall (%)	Precision (%)	F1-Score
Baseline (No augmentation)	73.21	76.87	83.70	0.8014
ReShuffle-MS	**83.25**	**97.28**	82.18	**0.8910**
Gaussian Noise Injection	72.25	72.11	**86.18**	0.7852
TIC Disturbance	76.56	82.99	83.56	0.8328

**Table 5:** Effect of region partitioning strategy on classification performance. All other settings kept constant. All models trained using CNN (MSDeepAMR). Bold indicates the best result.

Strategy	Accuracy (%)	Recall (%)	Precision (%)	F1-Score	AUROC
Shuffle-All	77.99	89.80	80.98	0.8516	0.8174
ReShuffle-MS	**83.25**	**97.28**	82.18	**0.8910**	**0.8391**
Shuffle-MDR-only	71.77	74.83	**83.33**	0.7885	0.8110
MDR Removal	68.42	85.71	73.68	0.7925	0.6178

**Table 6:** An ablation study structured as a 2 × 2 validation matrix evaluating label consistency and positional correspondence.

	Intra-Class	Cross-Class
positional correspondence	ReShuffle-MS	Global Shuffle
without positional correspondence	Free-Bin Shuffle	Global Free-Bin Shuffle

**Table 7:** Effect of recombination scope on model performance. All other settings kept constant. All models trained using CNN (MSDeepAMR). Bold indicates the best result.

Strategy	Accuracy (%)	Recall (%)	Precision (%)	F1-Score	AUROC
ReShuffle-MS	**83.25**	**97.28**	82.18	**0.8910**	**0.8391**
Global Shuffle	73.68	77.55	83.82	0.8057	0.7970
Free-Bin Shuffle	77.99	85.03	**83.89**	0.8446	0.7992
Global Free-Bin Shuffle	77.03	85.03	82.78	0.8389	0.8036

**Table 8:** Effect of augmentation factor *k* on CNN (MSDeepAMR) performance under different data-limited training scenarios. Bold indicates the best result within each setting.

Training Set Size	Augmentation Factor (*k*)	Accuracy (%)	Recall (%)	Precision (%)	F1-Score
n=360	k=0	50.00	10.00	50.00	0.1667
	k=5	60.00	42.00	65.62	0.5122
	k=10	**64.00**	**52.00**	**68.42**	**0.5900**
n=500	k=0	70.33	77.55	79.72	0.7862
	k=5	75.60	80.27	**84.29**	0.8223
	k=10	**75.60**	**82.99**	82.43	**0.8271**

**Table 9:** Performance comparison on ceftriaxone resistance prediction in *E. coli* using the DRIAMS-C dataset. Dashes (–) indicate that the corresponding metric was not reported for that specific experiment in the original publication.

Method	Training Dataset	Testing Dataset	Accuracy (%)	AUROC	AUPRC
ReShuffle-MS (This study)	DRIAMS-C	DRIAMS-C	84.15	0.8584	0.5356
Weis et al. (2022) [23]	DRIAMS-C	DRIAMS-C	–	0.66	–
Weis et al. (2022) [23]	DRIAMS-A/B/C/D	DRIAMS-C	–	0.40 ± 0.06	–
Weis et al. (2022) [23]	DRIAMS-A	DRIAMS-A	–	0.74 ± 0.02	0.30 ± 0.03

## Data Availability

The datasets and the models used in this study are available in the Zenodo repository at https://doi.org/10.5281/zenodo.17139697.

## Supplementary Materials

The following supporting information can be downloaded at: https://www.mdpi.com/article/10.3390/microorganisms14010177/s1, Figure S1: Spectral similarity between resistant and susceptible isolates and the feature importance profile used to define the MDR. Figure S2: Effect of region partitioning parameters (α, *N*) and augmentation factor (*k*) on model performance (mean ± std). Figure S3: Case-level Grad-CAM visualizations for two representative test samples. Figure S4: t-SNE visualization of CNN embeddings for species-level analysis. Table S1: Hyperparameter search space used for grid search during model selection. Table S2: Species-level validation of original and augmented spectra using an independent CNN classifier.
