# Supplementary material for: ReShuffle-MS: Region-Guided Data Augmentation Improves Artificial Intelligence-Based Resistance Prediction in Escherichia coli from MALDI-TOF Mass Spectrometry
Source: Microorganisms. 2026 Jan 13;14(1):177. doi: 10.3390/microorganisms14010177 (PMC12844117; doi:10.3390/microorganisms14010177)
Supplement: Supplementary file 1 [file microorganisms-14-00177-s001.zip › microorganisms-4075156-supplementary.pdf]

Supplementary Materials

ReShuffle-MS: Region-Guided Data Augmentation Improves Artificial Intelligence-Based Resistance Prediction in *Escherichia coli* from MALDI-TOF Mass Spectrometry

Dongbo Dai; Chenyang Huang; Junjie Li; Xiao Wei; Shengzhou Li; Qiong Wu; Huiran Zhang

(A)

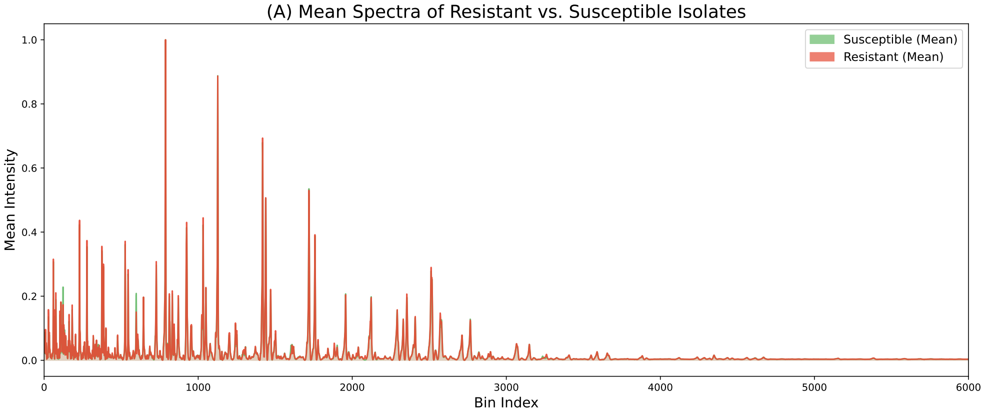

(B)

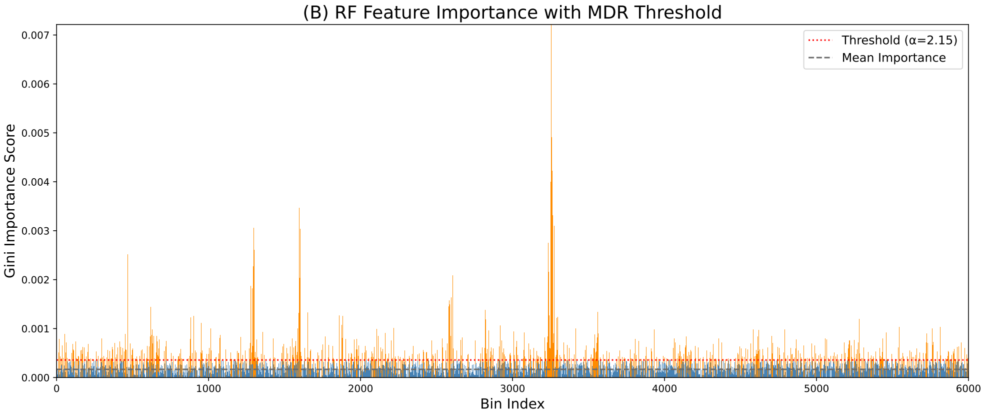

Figure S1. Spectral similarity between resistant and susceptible isolates and the feature importance profile used to define MDR. (A) Mean spectra of resistant and susceptible *E. coli* isolates: comparison of mean MALDI-TOF mass spectra for resistant (red) and susceptible (green) isolates. Despite minor differences in peak intensities, the overall spectral patterns are highly similar between the two classes. **(B)** Feature importance scores from Random Forest: each bin along the x-axis represents a spectral feature (bin), and the y-axis denotes its Gini importance score. The red dashed line marks the selection threshold ($\alpha = 2.15$ times the mean importance, shown as the grey dotted line). Bins above this threshold are designated the MDR (orange), and the remaining bins the PPR (blue).

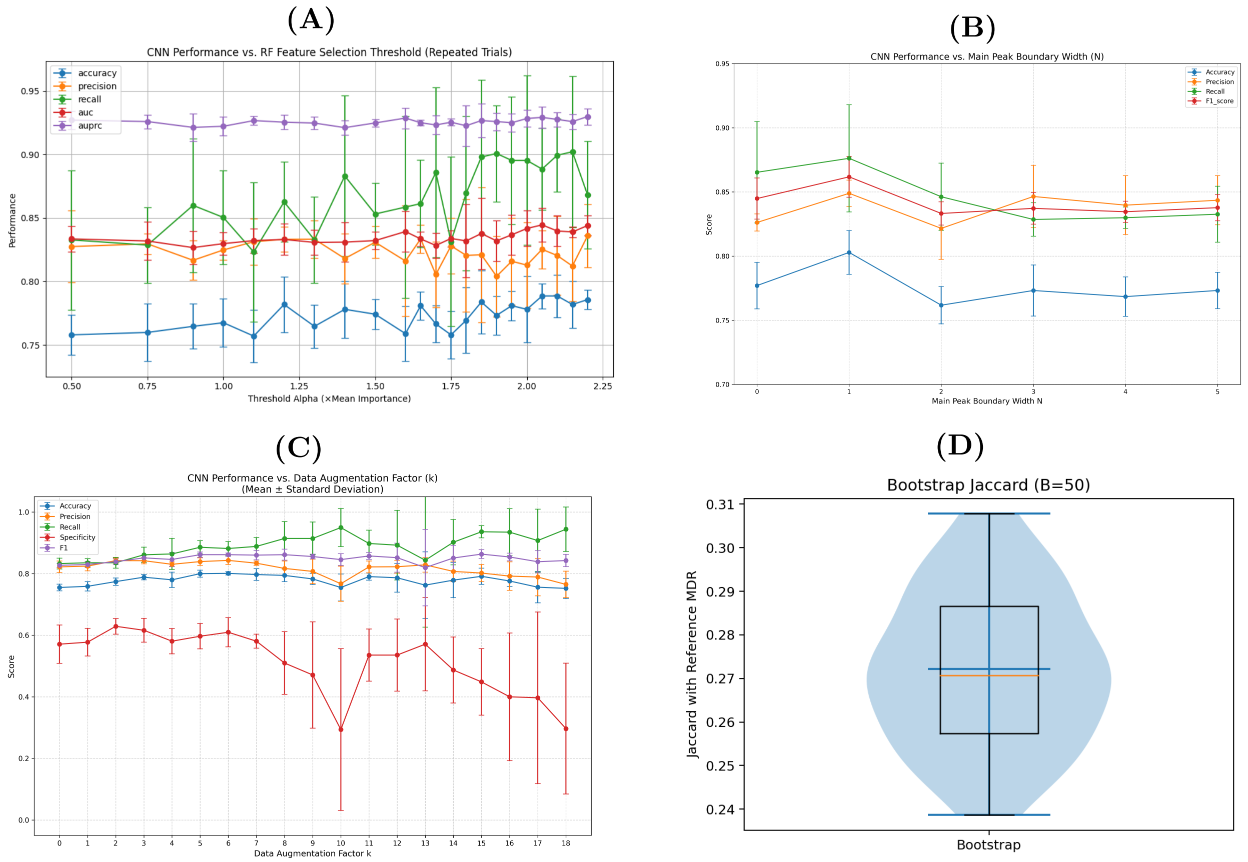

Figure S2. Effect of region partitioning parameters (α, N) and augmentation factor (k) on model performance (mean $\pm$ std). The CNN model used is MSDeepAMR. Error bars represent the standard deviation over five runs. **(A)** Model accuracy as a function of the main region threshold α. $\alpha \in$ [0.5, 0.75, 0.9, 1.0, 1.1, 1.2, 1.3, 1.4, 1.5, 1.6, 1.65, 1.7, 1.75, 1.8, 1.85, 1.9, 1.95, 2.0, 2.05, 2.1, 2.15, 2.2], N=0. With the increase of α, classification accuracy fluctuates and improves. Model performance remains stable across a broad range of α values. Considering higher recall, α = 2.15 was selected for all other experiments, unless otherwise specified. **(B)** Model accuracy as a function of the boundary size N. $N \in \{0, 1, 2, 3, 4, 5\}$, α=2.15. All metrics peak at N=1. Setting N=0 (only high-importance bins) is suboptimal, underscoring the benefit of preserving neighboring bins around high-importance bins. Increasing N to 2 or 3 yields no additional gain and slightly degrades performance, likely due to inclusion of less relevant adjacent regions into the MDR. We therefore use N=1 for all other experiments, unless otherwise specified. **(C)** Model performance as a func-tion of the augmentation factor k with α=2.15 and N=1 fixed. We evaluate $k \in \{0, 1, 2, \ldots, 18\}$. As k increases from 0 to 10, accuracy and recall steadily improve, indicating that moderate augmentation enhances generalization. Beyond k=8, specificity begins to fluctuate and decline sharply, suggesting potential overfitting or dilution of informative patterns due to excessive recombination. To balance sensitivity and specificity, we select k=6 for all other experiments, unless otherwise specified. **(D)** Bootstrap-based reproducibility of MDR identification (B=50). Violin and box plot show the distribution of Jaccard similarities between MDRs derived from bootstrap resamples and the reference MDR (mean $\pm$ sd = 0.272 $\pm$ 0.018).

Table S1. Hyperparameter search space used for grid search during model selection.

Model	Hyperparameters (grid search range)
LR	• penalty: [l1, l2]<br>• C: [0.001, 0.01, 0.1, 1, 10, 100]<br>• Note: `liblinear` solver was used to support both L1 and L2 penalties.<br>• *C* represents the regularization strength for Logistic Regression.
RF	• n_estimators: [100, 200]<br>• max_depth: [None, 10, 20]
SVM	• C: [0.01, 0.1, 1, 10, 100]<br>• kernel: [`linear`, `rbf`, `poly`]<br>• gamma: [`scale`, `auto`, 0.01, 0.001]<br>• degree: [2, 3]<br>• Note: *C* represents the regularization strength for SVM models.
MLP	• hidden layer sizes: [(512,), (1024,), (512, 256)]<br>• activation: [`relu`]<br>• learning_rate_init: [0.001]<br>• Note: `adam` solver was used.
XGBoost	• n_estimators: [70, 200]<br>• learning_rate: [0.01, 0.1]
LightGBM	• n_estimators: [100, 200]

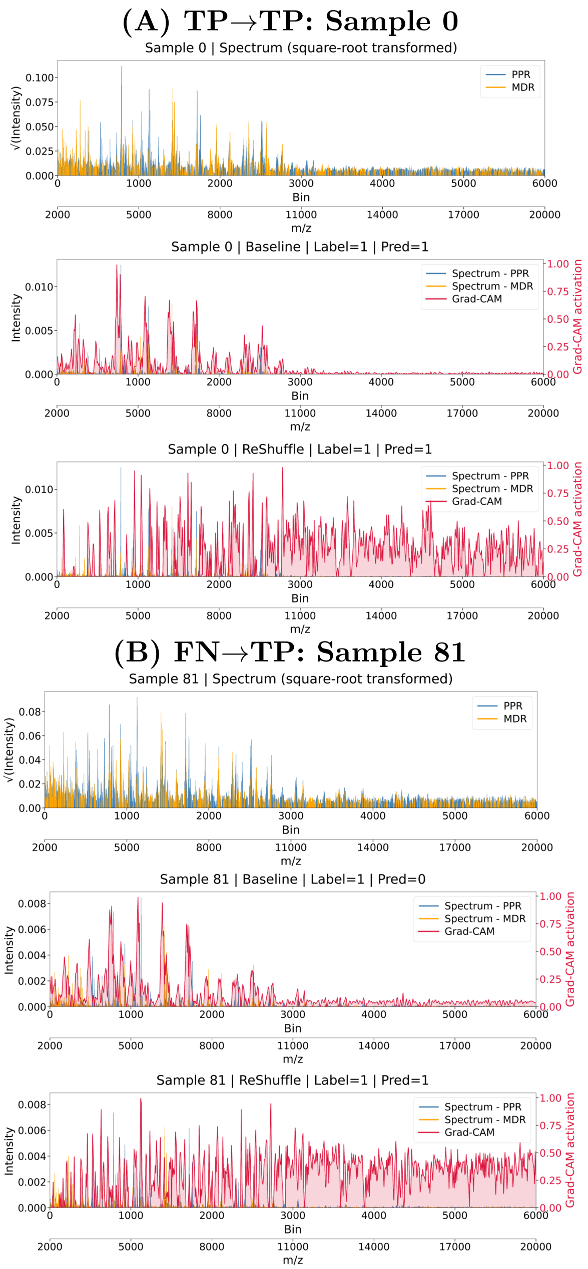

Figure S3. Case-level Grad-CAM visualizations for two representative test samples. In both cases, the spectrum is plotted with square-root transformed intensities to enhance the visibility of subtle peaks. PPR and MDR regions are marked in blue and orange, respectively. In both Grad-CAM visualization diagram, in order to be consistent with the model input, the original intensity data is retained. Grad-CAM activation maps are overlaid in red and reflect the raw model input scale. **(A)** Sample 0 (TP→TP): A resistant isolate that was correctly classified by both models. The ReShuffle-MS model exhibits a more evenly distributed attention pattern across the spectrum, extending into low-intensity and peripheral regions, while the baseline model shows sharply peaked attention concentrated in the early m/z region. This indicates that even when predictions are correct, ReShuffle-MS provides a more robust and interpretable basis for classification. **(B)** Sample 81 (FN→TP): A resistant isolate misclassified as sensitive by the baseline model but correctly classified by ReShuffle-MS. This confirms the framework's ability to recover relevant resistance-associated features, especially in cases where signal strength alone is not a sufficient discriminative cue.

Supplementary Analysis: Species-Level Validation of Augmented Spectra

To assess whether the proposed region-guided recombination strategy preserves species-level biological structure, we performed an independent species identification analysis using a CNN trained to classify three bacterial species.

We assembled a three-species MALDI-TOF MS dataset comprising 4,307 spectra, including *Staphylococcus aureus* (n = 479), *Escherichia coli* (n = 1,940), and *Klebsiella pneumoniae* (n = 1,888). The dataset was split into 80% for training and 20% for testing using stratified sampling to preserve class proportions in both subsets. Each spectrum was represented as 6,000 binned intensity features. Importantly, the preprocessing and normalization procedures were kept consistent with those used in the AMR prediction experiments, including the same binning scheme and normalization strategy.

A CNN species classifier was trained on the training set and evaluated on the held-out test set. The classification performance on the original test set is reported in Table S2A.

The trained species classifier was subsequently used to predict the species identity of the augmented *E. coli* spectra generated with an augmentation factor of $k = 6$. All augmented spectra were predicted as *E. coli* by the independent classifier (Table S2B), indicating that the region-guided recombination preserves species identity at the learned feature level.

In addition, t-SNE visualization was applied to CNN embeddings extracted from the fully connected layer to examine the distribution of original and augmented spectra in the learned representation space. As shown in Figure S4, augmented *E. coli* samples align closely with original *E. coli* spectra and remain well separated from other species.

Table S2. Species-level validation of original and augmented spectra using an independent CNN classifier.

(A) Performance of the CNN-based species classifier on the original test set.

Species	Precision	Recall	F1-score	Support
Staphylococcus aureus	0.9897	1.0000	0.9948	96
Escherichia coli	0.9923	0.9974	0.9949	388
Klebsiella pneumoniae	1.0000	0.9921	0.9960	378
**Accuracy**		0.9954		862
**Macro average**	0.9940	0.9965	0.9952	862
**Weighted average**	0.9954	0.9954	0.9954	862

(B) Species identification results for augmented *E. coli* spectra ($k = 6$).

Predicted species	Proportion (%)
Escherichia coli	100.00
Staphylococcus aureus	0.00
Klebsiella pneumoniae	0.00

(A) Original test set (three species)

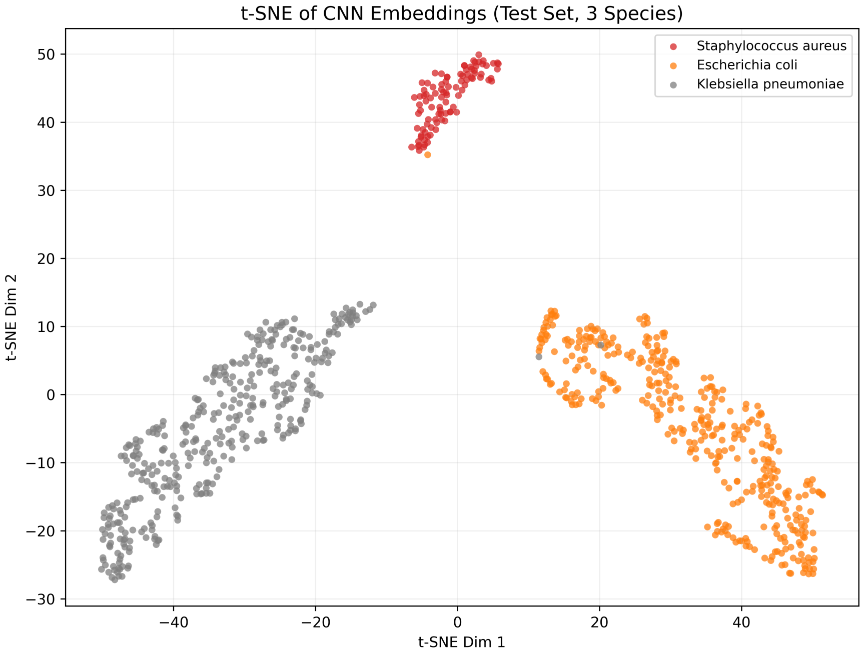

(B) Original species vs augmented *E. coli*

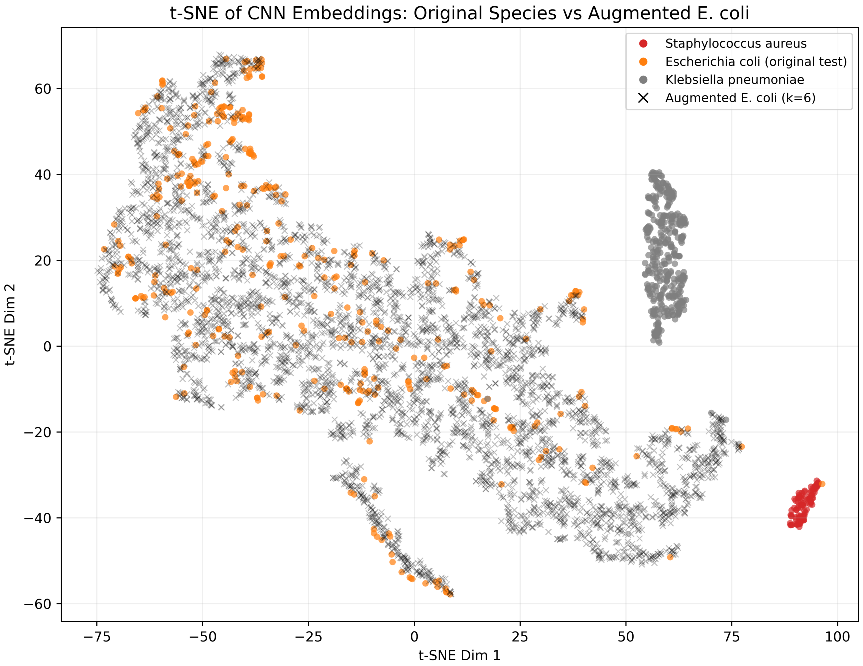

Figure S4. t-SNE visualization of CNN embeddings for species-level analysis. The embeddings were extracted from the fully connected layer of the CNN species classifier. **(A)** t-SNE projection of the original test set, showing clear separation among *Staphylococcus aureus*, *Escherichia coli*, and *Klebsiella pneumoniae*. **(B)** t-SNE projection of original test samples overlaid with augmented *E. coli* spectra (k = 6). Augmented samples (black crosses) largely overlap with the original *E. coli* cluster and remain well separated from other species, indicating that the region-guided recombination preserves species identity in the learned feature space.
